# Hypersplenism in Liver Cirrhosis: Is Conservative Treatment Still Best?

**DOI:** 10.1155/1995/67691

**Published:** 1995

**Authors:** Pavel G. Tarazov, Alexej A. Polykarpov

**Affiliations:** 1 Res. Inst. Roentgenol ul Leningradskaja 70/4 Pesochny-2 St. Petersburg 189646 Russia; 2 Division of Angio/Interventional St. Petersburg Research Institute of Roentgenology and Radiation Therapy St. Petersburg Russia


*HPB Surgery*, 1995, Vol. 9, pp. 55
Reprints available directly from the publisher
Photocopying permitted by license only

© 1995 OPA (Overseas Publishers Association)
Amsterdam B.V. Published in The Netherlands
by Harwood Academic Publishers GmbH
Printed in Malaysia

### Hyperspleenism in Liver Cirrhosis: Is Conservative Treatment Still Best?

PAVEL G. TARAZOV, and ALEXEJ A. POLYKARPOV

Division of Angio/Interventional, St. Petersburg Research, Institute of Roentgenology and Radiation Therapy, St. Petersburg, Russia

*(Received April 12, 1995)*

**LETTER TO THE EDITOR**

EDITOR — The best treatment for cirrhotic hypersplenism is still unknown. Both splenectomy and transcatheter splenic artery embolization have shown contradictory clinical results. We report our experience with the management of hypersplenism in 114 patients with liver cirrhosis.

The patients were divided in three groups in relation to treatment: I (splenectomy, 20 pts),II (proximal or peripheral splenic embolization, 48 pts), and III (conservative therapy, 46 pts). All groups were identical in sex, age, grade of hypersplenism (platelet count $36 \times 10^9$ /L to $75 \times 10^9$ /L), and Child-Pugh's Class of liver cirrhosis.

The results showed that splenectomy had rates of mortality and complications (5% and 10%) at least no higher than splenic embolization (10% and 19%). Improvement in hypersplenism to 1 year of follow-up was seen in 100% of patients in Group I vs 16% in Group II.

The survival rates did not differ significantly between all patients, however. In Groups I, II, and III, the 5-yr survival for Class A cirrhosis was 82%, 53%, and 81%, for Class B 70%, 30%, and 56%, respectively. The 3-yr survival rates for Class C cirrhosis were 33%, 14%, and 33%. The causes of death were identical in all groups, without prevalence of variceal bleeding among conservatively treated patients.

These results show that splenic embolization is more dangerous and less effective than traditional splenectomy for control of cirrhotic hypersplenism. However, both treatments do not prolong survival of patients if compared with conservative therapy.

Pavel G. Tarazov, MD
Alexej A. Polykarpov, MD
Division of Angio/Interventional,
St. Petersburg Research Institute of
Roentgenology and Radiation Therapy,
Pesochny-2, St. Petersburg, 189646 Russia.

*Address for correspondence:* P.G. Tarazov, MD Res Inst Roentgenol ul Leningradskaja 70/4 Pesochny-2 St Petersburg 189646 Russia Phone: (812) 437–8766 Fax: (812) 437-5600.

